# Quantum oscillations in a dipolar excitonic insulator

**DOI:** 10.1038/s41563-025-02334-3

**Published:** 2025-08-25

**Authors:** Phuong X. Nguyen, Raghav Chaturvedi, Bo Zou, Kenji Watanabe, Takashi Taniguchi, Allan H. MacDonald, Kin Fai Mak, Jie Shan

**Affiliations:** 1 School of Applied and Engineering Physics, Cornell University, Ithaca, NY USA; 2 Kavli Institute at Cornell for Nanoscale Science, Ithaca, NY USA; 3 Department of Physics, University of Texas at Austin, Austin, TX USA; 4 National Institute for Materials Science, Tsukuba, Japan; 5 Laboratory of Atomic and Solid State Physics, Cornell University, Ithaca, NY USA; 6 Max Planck Institute for the Structure and Dynamics of Matter, Hamburg, Germany

**Keywords:** Bose-Einstein condensates, Quantum fluids and solids, Quantum Hall, Two-dimensional materials

## Abstract

Quantum oscillations in magnetization or resistivity are a defining feature of metals in a magnetic field. The phenomenon is generally not expected in insulators without a Fermi surface. Its observation in Kondo and other correlated insulators provided counterexamples and remains poorly understood. Here we report the observation of resistivity oscillations in a gate-controlled excitonic insulator realized in Coulomb-coupled electron–hole double layers. When the electron or hole cyclotron energy is tuned to exceed the exciton binding energy, recurring transitions arise between the excitonic insulator and layer-decoupled quantum Hall states. Compressibility measurements show an oscillatory exciton binding energy as a function of the magnetic field and electron–hole pair density. Coulomb drag measurements further reveal the signature of finite-angular-momentum excitonic correlations. These findings are qualitatively captured by mean-field calculations. Our study establishes a highly tunable platform based on electron–hole double layers for studying quantum oscillations in correlated insulators.

## Abstract (web summary)

Quantum oscillations are reported in Coulomb-coupled electron–hole double layers that originate from recurring transitions between competing excitonic insulator and layer-decoupled quantum Hall states.

## Main

The observation of quantum oscillations in magnetization or resistivity in insulators^1–10^ has intrigued the condensed-matter physics community because it is long believed that the phenomenon could occur only in metals with a well-defined Fermi surface. Many theoretical proposals have been put forth to explain the observation, including magnetic-field-induced gap oscillations in both single-particle and correlated insulators^11–22^, competing many-body ground states as a function of the magnetic field^18,19,21,22^ and an exotic fractionalization scenario with neutral fermions and gauge fields^23,24^. So far, the mechanism responsible for oscillations in different material systems remains under debate, especially for strongly correlated systems. Limits on the ability to control the properties of many of the bulk correlated materials have hindered the development of a cohesive understanding. The emergence of highly tunable correlated insulating states in van der Waals’ heterostructures provides an opportunity to study this problem from a fresh perspective.

In this work, we report the observation of quantum oscillations in the resistivity, exciton binding energy and exciton current in a dipolar excitonic insulator (EI)^25–32^. This is achieved through a suite of magnetotransport, capacitance and drag counterflow measurements performed on Coulomb-coupled MoSe_2_/WSe_2_ electron–hole double layers. The quantum oscillations originate from recurring transitions between competing EI and layer-decoupled quantum Hall (QH) states as a function of the magnetic field and electron–hole pair density^19,21,22^. Compared with earlier reports on quantum oscillations in EI candidate InAs/GaSb quantum wells^6–8,33,34^, the ability to electrically tune and address the electron and hole layers separately has allowed us to establish the emergence of quantum oscillations in an EI.

## Electron–hole double layer

**Fig. 1:**
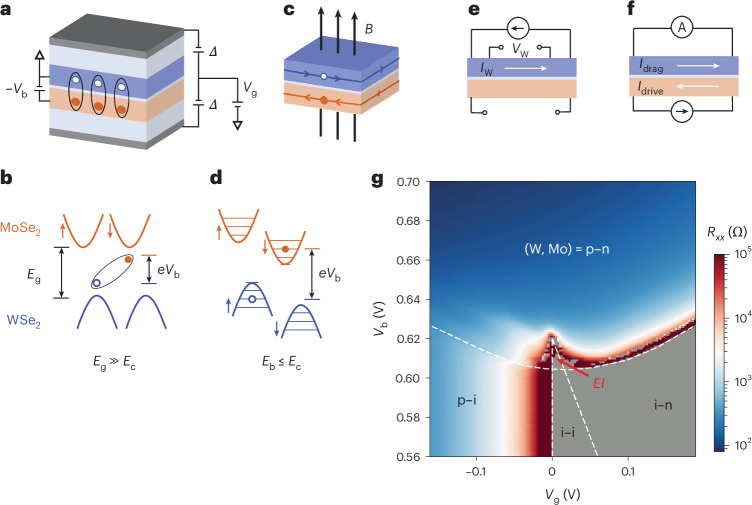
Coulomb-coupled MoSe_2_/WSe_2_ double layer. **a**, Device schematic. Interlayer bias voltage *V*_b_ (with the W layer grounded), *V*_g_ and *Δ* control the exciton chemical potential, net charge density and out-of-plane electric field in the double layer, respectively. Interlayer excitons (oval) are stabilized at *B* = 0 T and low electron–hole pair densities. Blue, WSe_2_ monolayer; orange, MoSe_2_ bilayer; light blue, hBN; grey, graphite. **b**, Band alignment under *B* = 0 T or for the exciton binding energy *E*_b_ far exceeding the cyclotron energy *E*_c_ (layers not angle aligned). Conduction bands are shown in orange, and valence bands, in blue. Arrows, spin states; dashed lines, Fermi levels. Interlayer excitons spontaneously form when *E*_b_ exceeds the effective charge gap (*E*_g_ – *eV*_b_). **c**, Same data as **a** but for high magnetic fields and high pair densities. Layer-decoupled QH states with counterpropagating electron and hole chiral edge states emerge. **d**, Same data as **b** but for *E*_b_ ≤ *E*_c_. Electron and hole LLs (horizontal solid lines) are spin-split by the Zeeman effect. **e**,**f**, Schematic of the open-circuit measurements of *R*_*xx*_ and *R*_*xy*_ in the W layer (**e**) and drag counterflow measurements (**f**), where *I*_W_ and *V*_W_ in **e** denote the current and induced voltage, respectively, and *I*_drive_ and *I*_drag_ in **f** are the drive current in the Mo layer and the induced drag current in the W layer, respectively. **g**, *R*_*xx*_ as a function of *V*_g_ and *V*_b_ at *B* = 0 T and *T* = 1.5 K. The dashed lines separate the EI and different doping regions with i, p and n, denoting the intrinsic, hole-doped and electron-doped layers, respectively. In the grey area, *R*_*xx*_ diverges and cannot be reliably determined. Source data

**Extended Data Fig. 1:**
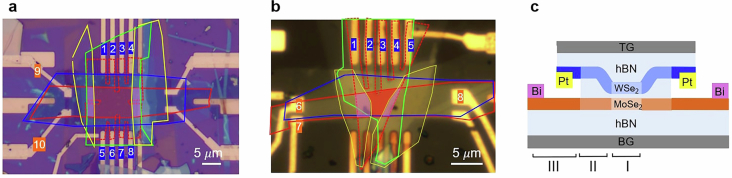
Device images. **a**,**b**, Optical micrographs for device 1 (**a**) and 2 (**b**). The top gate (red dashed line), bottom gate (red line), W-layer (green line), Mo-layer (blue line) and the exciton contact hBN layers (yellow lines) are outlined. The red- and pink-shaded areas denote the device channel and exciton contact regions, respectively. The contact electrodes to the Mo- and W-layers are numbered in orange and blue, respectively. **c**, Schematic cross section of the devices showing the doping profile. Region I, II and III are the channel, the exciton contact and the metal-semiconductor contacts, in increasing order of doping concentration and darkness of the color (orange for electron doping, blue for hole doping). TG, BG, Pt and Bi denote top gate, bottom gate, platinum electrode and bismuth electrode, respectively.

**Extended Data Fig. 2:**
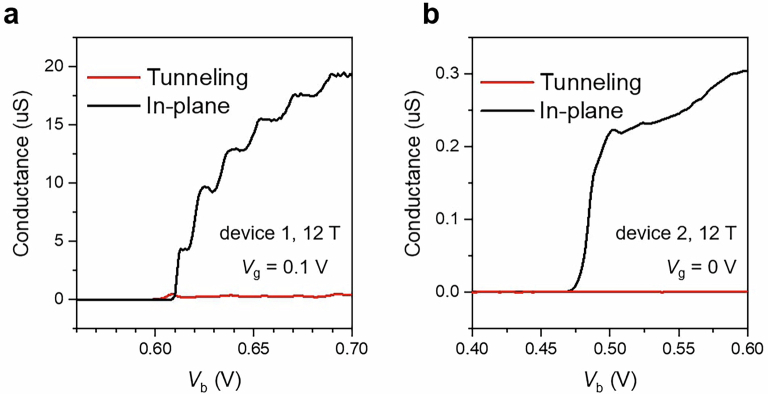
Negligible interlayer tunneling conductance. **a,b**, Interlayer tunneling conductance (red) and two-terminal in-plane conductance of the Mo-layer (black) as a function of ${V}_{b}$ for device 1 at ${V}_{g}=0.1$ V **(a)** and device 2 at ${V}_{g}=0$ V **(b)**. In the relevant *pn* and EI regions (that is when the Mo-layer is turned on electrically), both devices show tunneling conductance about 1-2 orders of magnitude smaller than the in-plane conductance.

Figure 1a shows a schematic of the cross-section of a dual-gated electron–hole double-layer device. The double layer consists of a natural MoSe_2_ bilayer and a WSe_2_ monolayer separated by a thin hexagonal boron nitride (hBN) barrier (5–6 layers thick; Extended Data Fig. 1; the MoSe_2_ bilayer is more robust for fabrication but behaves practically like a monolayer because electrons are polarized to one layer under a high perpendicular electric field in this study). The MoSe_2_/WSe_2_ heterobilayer has a type-II band alignment^26–30,32^ (Fig. 1b), with the conduction (valence) band minimum (maximum) residing in the Mo layer (W layer). The two gates allow for independent control of the net electron doping density (*n*) in the double layer and the electric field (*E*) perpendicular to the sample plane through the symmetric (*V*_g_) and antisymmetric (*Δ*) parts of the two gate voltages (for symmetric gates), respectively. We apply a constant electric field to reduce the heterobilayer energy gap (from *E*_g_ ≈ 1.6 eV (refs. ^27,29,32^) to *E*_g_ ≈ 0.625 eV in device 1). We also apply a bias voltage (*V*_b_) between the Mo and W layers to separate the electron and hole Fermi levels. When the effective charge gap of the double layer (*E*_g_ – *eV*_b_) becomes smaller than the interlayer exciton binding energy (*E*_b_), the double layer is spontaneously populated by interlayer excitons, forming an EI with a net electric polarization^35–38^ (that is, a dipolar EI). Here *e* is the elementary charge. The electrons in the Mo layer and holes in the W layer are coupled only through the interlayer Coulomb interactions. With negligible single-particle interlayer tunnelling (verified by negligible interlayer tunnelling currents (Extended Data Fig. 2)), the excitons in the double layers are in thermal equilibrium with an exciton chemical potential (*eV*_b_ – *E*_g_). Note that the EI here refers only to a charge insulating state and does not imply spontaneous interlayer coherence.

In the presence of a strong perpendicular magnetic field *B*, Landau levels (LLs) emerge in the electron and hole layers (Fig. 1c,d). When the electron–hole pair density (*n*_p_) exceeds the exciton Mott density (*n*_M_), the excitons dissociate into an electron–hole plasma^27,29,35,36^, and layer-decoupled QH states are expected. On the other hand, for low pair densities and low magnetic fields, when the electron–hole cyclotron energy (*E*_c_) is comparable to *E*_b_, EI is expected to dominate (the electron and hole cyclotron energies are comparable in MoSe_2_/WSe_2_). In the interesting regime of *n*_p_ ≈ *n*_M_ and *E*_c_ ≈ *E*_b_, it was suggested in refs. ^21,22^ that recurring transitions between the EI and layer-decoupled QH states could emerge as a function of *n*_p_ and *B*. Unlike the interlayer-coherent exciton condensates in half-filled LLs in electron–electron or hole–hole Coulomb-coupled bilayers^39–43^, in which the condensates carry chiral edge states (that is, they are QH ferromagnets) and are unstable in the zero-field limit^21^, the EI in the electron–hole double layers is stable in the zero-field limit and does not support chiral edge states because interlayer coherence is expected to gap out the counterpropagating edge states^21,22^.

**Extended Data Fig. 3:**
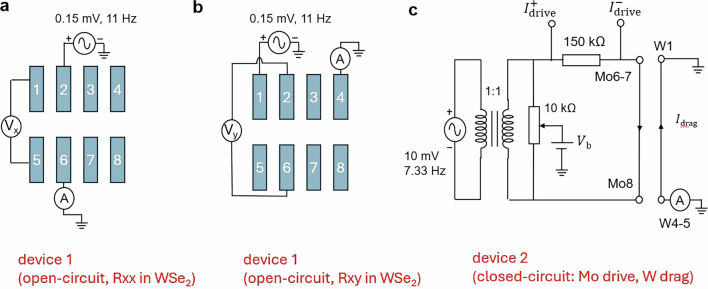
Measurement circuit diagrams. **a,b**, Open-circuit measurement diagram for ${R}_{{xx}}$ (**a**) and ${R}_{{xy}}$ (**b**) in device 1. Only the pins in the W-layer are shown. The Mo-layer is in open-circuit; only pin 9 (one of the Mo-layer contacts) is connected to apply a DC interlayer bias ${V}_{b}$. **c**, Closed-circuit measurement diagram for the drag counterflow studies in device 2. An AC electron current is biased in the Mo-layer through a 1:1 transformer. ${V}_{b}$ is applied (through the midpoint of a 10 kΩ potentiometer) to both the source (shorted pins 6 and 7, Mo6-7) and drain (pin 8, Mo8) in the Mo-layer. The potentiometer position is tuned to minimize the AC interlayer coupling. In the W-layer, pin 1 (W1) is grounded and the hole drag current is measured by an ammeter connected to shorted pin 4 and 5 (W4-5).

We probe the double layer under finite magnetic fields by patterning it into a Hall bar device with independent electrical contacts to the Mo and W layers. Because the electron contacts formed via bismuth evaporation in the Mo layer are less reliable, we measure the four-terminal longitudinal (*R*_*xx*_) and Hall (*R*_*xy*_) resistances in the W layer and keep the Mo layer in the open-circuit condition (Fig. 1e and Extended Data Fig. 3). As demonstrated by a recent study^32^, *R*_*xx*_ and *R*_*xy*_ are highly sensitive to interlayer Coulomb interactions. This measurement is supplemented by the drag counterflow measurement (Fig. 1f), which has been shown to directly probe the interlayer Coulomb interactions^29,30,41^ and the penetration capacitance measurement^27^, which accesses the exciton binding energy in the EI^27,38^. Methods provides details on the device fabrication and electrical transport and capacitance measurements. Unless otherwise specified, the sample temperature is *T* = 1.5 K.

## Resistivity oscillations in EI

Figure 1g shows *R*_*xx*_ of the W layer as a function of *V*_g_ and *V*_b_ at *B* = 0 T (device 1), where the gate voltage *V*_g_ tunes the net doping density *n* in the double layer and the bias voltage *V*_b_ mainly controls the electron–hole pair density *n*_p_ (ref. ^27^). The electrostatic phase diagram shows i–i, i–n, p–i and p–n regions for the (W, Mo) layers, where i, p and n denote an intrinsic (or charge-neutral), hole-doped and electron-doped layer, respectively. As expected, *R*_*xx*_ diverges at low temperature in the i–i and i–n regions (grey shaded); the W layer is conducting only in the p–i and p–n regions (the p–i*|*i–i boundary is pinned to *V*_g_ = 0 because the W layer is grounded). *R*_*xx*_ also diverges in the EI region (the triangular i–i region that protrudes into the p–n region), in which a hole current in the W layer is not allowed because the electrons and holes are bound and the electron layer is in the open-circuit condition^32^. At the tip of the EI triangle, the pair density reaches the exciton Mott density, and the interlayer excitons dissociate into an electron–hole plasma^27,29^. The results are fully consistent with earlier studies^27,29,32^.

**Fig. 2:**
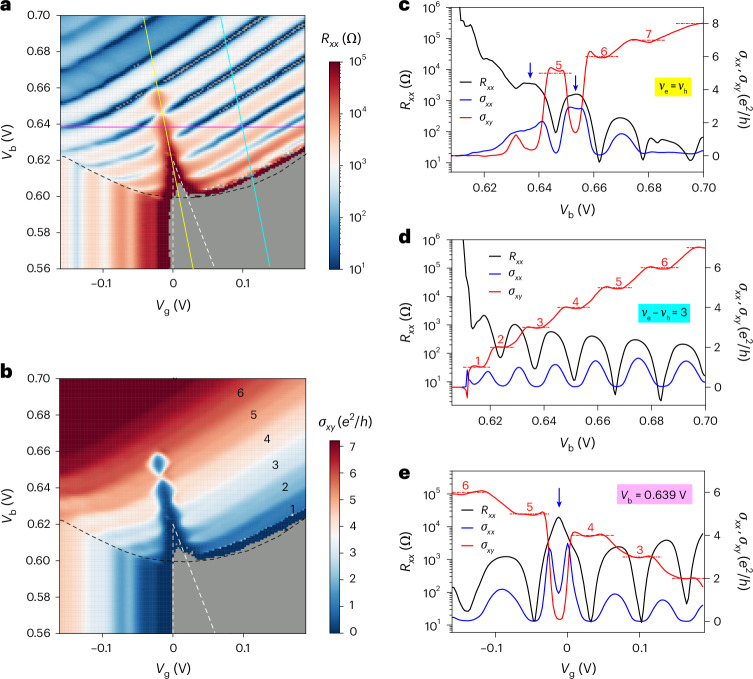
Quantum oscillations in the EI phase. **a**,**b**, *R*_*xx*_ (**a**) and *σ*_*xy*_ (**b**) of the W layer (with the Mo layer in the open-circuit configuration) as a function of *V*_g_ and *V*_b_ at *B* = 12 T and *T* = 1.5 K. The black and white dashed lines are the phase boundaries from the data for *B* = 0 in Fig. 1a. The EI phase is shifted and expanded by the magnetic field. Quantized ${\sigma }_{{xy}}=\frac{{\nu }_{\rm{h}}{e}^{2}}{h}$ for *ν*_h_ = 1–6 is identified in **b**. In the grey area, *R*_*xx*_ and *σ*_*xy*_ cannot be reliably measured because of the diverging *R*_*xx*_. **c**–**e**, *R*_*xx*_ (left axis) and *σ*_*xx*_ and *σ*_*xy*_ (right axis) along the solid yellow (**c**), cyan (**d**) and purple (**e**) lines in **a**. The QH states are characterized by *R*_*xx*_ and *σ*_*xx*_ dips and quantized *σ*_*xy*_ (horizontal dashed lines). The EI states are characterized by *R*_*xx*_ peaks and suppressed *σ*_*xx*_ and *σ*_*xy*_. Blue arrows identify strong EI states between two integer QH states. Source data

**Extended Data Fig. 4:**
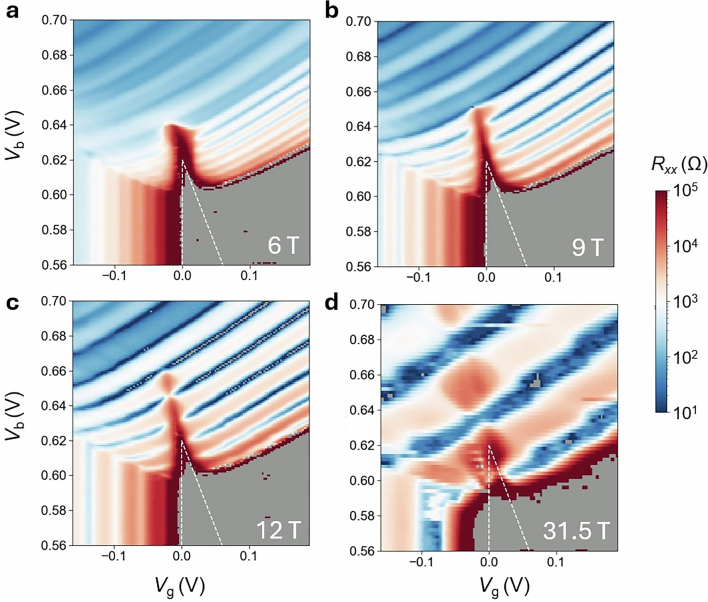
Magnetic-field evolution of *R*_*xx*_*.* **a-d**, ${R}_{{xx}}$ of the W-layer (with the Mo-layer in open-circuit) as a function of ${V}_{g}$ and ${V}_{b}$ at $B=6$ T (**a**), 9 T (**b**), 12 T (**c**) and 31.5 T (**d**). The dashed lines locate the band edges under zero magnetic field. ${R}_{{xx}}$ diverges and cannot be reliably measured in the grey area. The dips in ${R}_{{xx}}$ reflect the fully filled LLs. The voltage spacing between consecutive dips increases with $B$ as the LL degeneracy $\Delta {n}_{{LL}}=\frac{{eB}}{h}$ increases. At $B=31.5$ T, the lowest LL penetrates through the triangular EI region.

**Extended Data Fig. 5:**
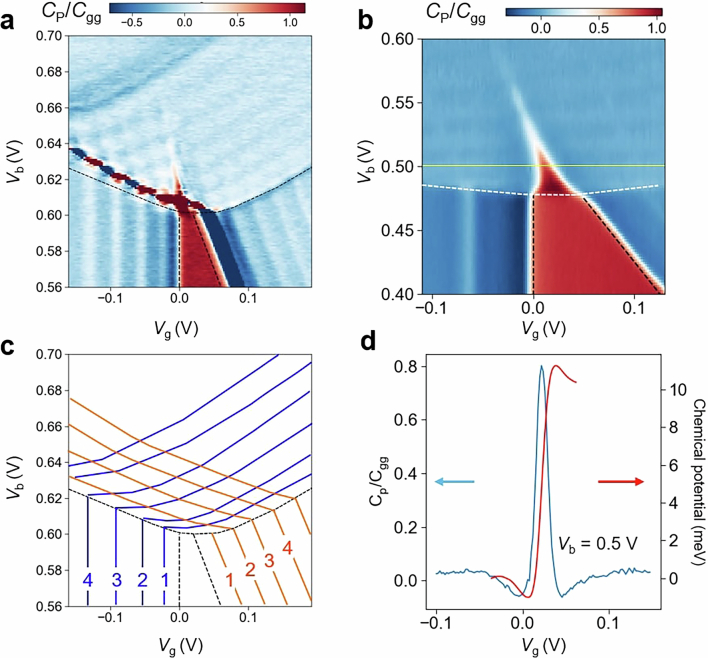
Penetration capacitance measurements. **a,b**, The normalized penetration capacitance ${C}_{{\rm{P}}}/{C}_{{\rm{gg}}}$ as a function of ${V}_{g}$ and ${V}_{b}$ at $B=12$ T and $T=1.5$ K for device 1 **(a)** and 2 **(b)**. LLs from both the W- and Mo-layers can be observed, especially in device 1. The electronic incompressibility of the EI oscillates as ${V}_{b}$ increases at charge-neutrality. **c**, Integer-filled LLs for the Mo- (orange) and W-layer (blue) extracted from the data in **a**. **d**, ${C}_{{\rm{P}}}/{C}_{{\rm{gg}}}$ (blue, left axis) and the extracted electron chemical potential (red, right axis) as a function of ${V}_{g}$ at ${V}_{b}$ = 0.5 V (yellow solid line in **b**). The chemical potential is obtained by integrating ${C}_{{\rm{P}}}/{C}_{{\rm{gg}}}$ with respect to ${V}_{g}$ (see Methods). The exciton binding energy is equal to the jump in the chemical potential for the incompressible EI state.

Next, we examine *R*_*xx*_ and *σ*_*xy*_ (Hall conductivity) as a function of *V*_g_ and *V*_b_ at *B* = 12 T (Fig. 2a,b). Results at other magnetic fields up to 31.5 T are shown in Extended Data Fig. 4. Hole LLs are observed in the p–i and p–n regions. Each fully filled LL is characterized by an *R*_*xx*_ dip and a nearly quantized *σ*_*xy*_ at $\frac{{\nu }_{\rm{h}}{e}^{2}}{2\uppi \hslash }$ (see the line cuts in Fig. 2d). Here *V*_b(e)_ is the hole (electron) LL filling factor and *ℏ* is the reduced Planck constant. In the p–i region, the hole LLs form vertical stripes because the hole density is independent of *V*_b_. In the p–n region, they form diagonal stripes because the doping density in the W layer (and the Mo layer) changes with both *V*_g_ and *V*_b_. The electron LLs in the Mo layer are nearly invisible in *R*_*xx*_ and *σ*_*xy*_ of the W layer; they are better resolved in the drag counterflow (Fig. 5) and capacitance (Extended Data Fig. 5) measurements.

The most interesting features in the *R*_*xx*_ and *σ*_*xy*_ maps are located near the EI triangle of the phase diagram. At the bottom of the triangle (0.60 ≲ *V*_b_ ≲ 0.64 V), where the exciton binding energy is large, the diagonal LL stripes are interrupted by the EI with diverging *R*_*xx*_ and vanishing *σ*_*xy*_ (only at the highest magnetic fields (Extended Data Fig. 4), the LL stripes penetrate through the EI). Near the tip of the triangle (0.64 ≲ *V*_b_ ≲ 0.66 V), where the exciton binding energy is small, a fully filled LL can nearly penetrate through the EI without interruption even under moderate magnetic fields; on the other hand, a half-filled LL (one with an *R*_*xx*_ peak) transitions to the EI with much higher *R*_*xx*_ and strongly suppressed *σ*_*xy*_.

Figure 2c–e shows three representative line cuts along the solid lines shown in the maps (the extracted longitudinal conductivity *σ*_*xx*_ is also included). Only a series of QH states are observed away from charge neutrality at *ν*_e_ − *ν*_h_ = 3 (Fig. 2d). At charge neutrality (Fig. 2c), the transition from the *ν*_h_ = 6 QH state (*V*_b_ ≈ 0.662 V) to the *ν*_h_ = 5 QH state (*V*_b_ ≈ 0.645 V) is interrupted by a weak EI near *V*_b_ ≈ 0.653 V; it shows enhanced *R*_*xx*_ and suppressed *σ*_*xy*_ and *σ*_*xx*_ (on top of the wide peak). Only the EIs with diverging *R*_*xx*_ and vanishing *σ*_*xx*_ and *σ*_*xy*_ are stable (*V*_b_ ≲ 0.64 V). In Fig. 2e, an EI with a sharp dip in *σ*_*xx*_ near *V*_g_ ≈ –0.01 V, which interrupts the transition from the *ν*_h_ = 5 QH state (*V*_g_ ≈ –0.05 V) to the *ν*_h_ = 4 QH state (*V*_g_ ≈ 0.03 V), is also illustrated by a line cut at constant *V*_b_ ≈ 0.64 V.

**Fig. 3:**
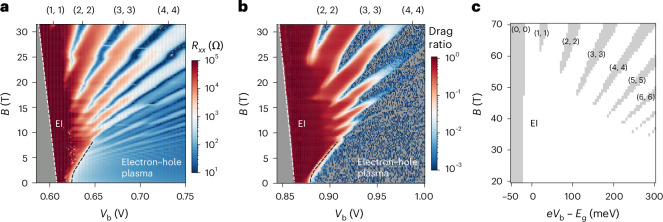
Magnetic-field phase diagram at charge neutrality. **a**,**b**, *R*_*xx*_ of the W layer (device 1; **a**) and drag ratio $\frac{{I}_{\rm{drag}}}{{I}_{\rm{drive}}}$ (device 3; **b**) as a function of *V*_b_ and *B* at charge neutrality and *T* = 0.3 K. The white dashed line denotes the band edge, and the black dashed line separates the EI and the electron–hole plasma phases. In the grey area (left of the white line), *R*_*xx*_ diverges and cannot be reliably measured. The EI phase with diverging *R*_*xx*_ and large $\frac{{I}_{\rm{drag}}}{{I}_{\rm{drive}}}$ expands with the magnetic field. The fully filled LLs with vanishing *R*_*xx*_ and $\frac{{I}_{\rm{drag}}}{{I}_{\rm{drive}}}$ and (*ν*_e_, *ν*_h_) = (1, 1), (2, 2), (3, 3), (4, 4) protrude into the EI phase. **c**, Mean-field phase diagram in exciton chemical potential (*eV*_b_ – *E*_g_) and *B* at charge neutrality (Methods). White region, EI; grey, layer-decoupled QH states. Source data

We examine the magnetic-field dependence of *R*_*xx*_ at charge neutrality in Fig. 3a (along the yellow solid line (Fig. 2a)) up to *B* = 31.5 T at 0.3 K. With increasing field, the EI region expands. On one hand, the exciton Mott transition or the metal–insulator transition (black dashed line) moves to higher *V*_b_ in a hyperbolic fashion due to exciton diamagnetic shift^44^. On the other hand, the onset for exciton injection (white dashed line) shifts linearly to lower *V*_b_ due to the exciton Zeeman effect. We obtain an interlayer exciton *g*-factor of ~20, in agreement with earlier optical studies^45^. LLs emerge in the electron–hole plasma region for *B* ≳ 3T. They disperse with *V*_b_ slightly nonlinearly because the interlayer capacitance depends on *V*_b_ (ref. ^27^). At high fields, the EI phase with large *R*_*xx*_ is interrupted by LLs with *R*_*xx*_ dips (*ν*_e_ = *ν*_h_ = 1, 2, 3 and 4). The onset field for the LLs decreases with increasing *ν*_e_ or *ν*_h_. The distinct horizontal stripes in the phase diagram are caused by the LLs in the graphite gates, which are capacitively coupled to the electron–hole double layer^9,10^; they are independent of *V*_b_ because the W layer is grounded.

**Fig. 4:**
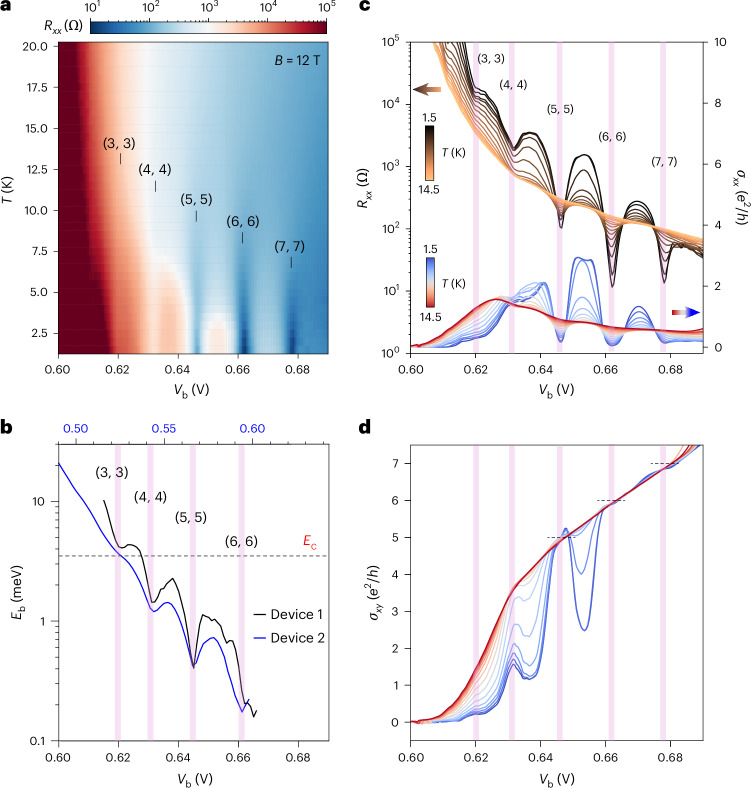
Oscillating exciton binding energy (*B* = 12 T). **a**, *R*_*xx*_ of the W layer as a function of *V*_b_ and *T* at charge neutrality. **b**, Exciton binding energy as a function of *V*_b_ (bottom axis for device 1 and top axis for device 2). It oscillates on a monotonically decreasing background. The horizontal dashed line marks the cyclotron energy *E*_c_ (comparable for electrons and holes). **c**,**d**, *R*_*xx*_ (left axis; **c**), *σ*_*xx*_ (right axis; **c**) and *σ*_*xy*_ (**d**) versus *V*_b_ at varying temperatures (1.5 K to 14.5 K in 1-K step) for device 1. The dashed lines in **d** denote the quantized values. As *V*_b_ increases, the EI state (with increasing *R*_*xx*_ and decreasing *σ*_*xx*_ and *σ*_*xy*_) transitions into the QH states (with decreasing *R*_*xx*_ and *σ*_*xx*_ and quantized *σ*_*xy*_) at low temperatures. The fully filled LLs (*ν*_e_ and *ν*_h_) are labelled in **a** and denoted by the vertical purple lines in **b**–**d**. Source data

**Extended Data Fig. 6:**
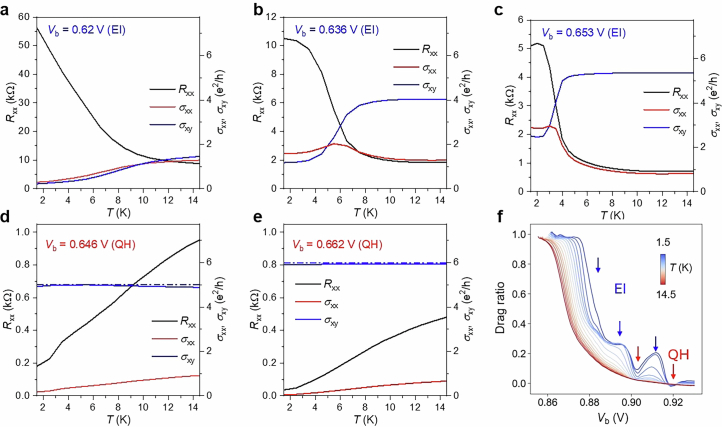
Temperature dependence of *R*_*xx*_, *σ*_*xx*_, *σ*_*xy*_ and drag ratio. **a-e**, Temperature dependence of ${R}_{{\rm{xx}}}$ (left axis), ${\sigma }_{{\rm{xx}}}$ and ${\sigma }_{{\rm{xy}}}$ (right axis) (device 1) at ${\nu }_{e}={\nu }_{h}$ for the EI at ${V}_{b}=$ 0.62 V (**a**), 0.636 V (**b**) and 0.653 V (**c**) and for the QH states at ${V}_{b}=$ 0.646 V (**d**) and 0.662 V (**e**). Blue dashed-dotted lines in **d,e** mark the expected quantized values for ${\sigma }_{{\rm{xy}}}$. **f**, Drag current ratio (device 3) as a function of ${V}_{b}$ at ${\nu }_{e}={\nu }_{h}$ for temperature varying from 1.5 K to 14.5 K in 1 K step. Blue (red) arrows mark the EI (QH) states with enhanced (suppressed) drag current ratio.

**Extended Data Fig. 7:**
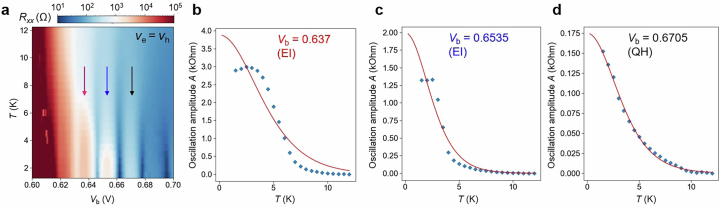
Temperature dependence of the quantum oscillation amplitude. **a**, ${R}_{{xx}}$ of the W-layer as a function of ${V}_{b}$ and $T$ at charge neutrality (copy of Fig. 4a of the main text). **b-d**, Temperature dependence of the oscillation amplitude (blue symbols) for three representative ${V}_{b}$’s marked by arrows in **a**: ${V}_{b}=0.637$ V **(b)**, ${V}_{b}=0.6535$ V **(c)** and ${V}_{b}=0.6705$ V **(d)**. The oscillation amplitude at each temperature was extracted by subtracting from the resistance ${R}_{{xx}}(T=12K)$, at which quantum oscillations are no longer observable. Solid red lines are the Lifshitz-Kosevich (LK) fits. Good agreement is observed for the QH state in **d**; the extracted effective mass is consistent with the reported mass for monolayer WSe_2_. Significant deviation from the LK formula is observed for the EI phase in **b** and **c**.

We further study the temperature dependence of the quantum oscillations at constant *B* = 12 T. Figure 4a shows *R*_*xx*_ as a function of *V*_b_ and temperature. Figure 4c displays line cuts at the representative temperatures. Figure 4c,d also includes *σ*_*xx*_ and *σ*_*xy*_ (Extended Data Fig. 6 shows additional data). Recurring transitions between the EI and QH states are observed as *V*_b_ increases. As the temperature decreases, *R*_*xx*_ increases, and *σ*_*xx*_ and *σ*_*xy*_ decrease for the EI states; *R*_*xx*_ and *σ*_*xx*_ decrease, and *σ*_*xy*_ approaches a quantized value for the QH states. At the boundary separating the two states, these quantities are nearly temperature independent. The temperature dependence of the oscillation amplitude of *R*_*xx*_ is analysed in Extended Data Fig. 7. It follows the Lifshitz–Kosevich formula for the QH states and substantially deviates from this formula for the EI states. Quantum oscillations are also observed in the penetration capacitance, from which we extract the exciton binding energy as a function of *V*_b_ (Methods and Extended Data Fig. 5). The results from two different devices (devices 1 and 2) in Fig. 4b are similar. The binding energy oscillates as a function of *V*_b_ on a monotonic decreasing background; the local minimum corresponds to a QH state.

**Extended Data Fig. 8:**
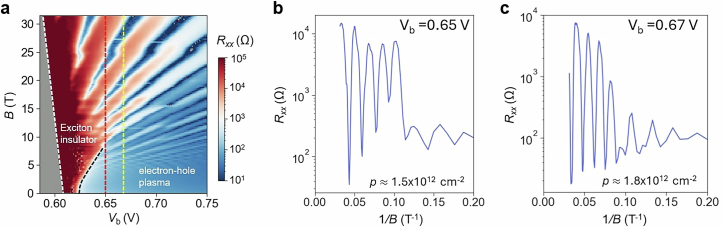
Periodic quantum oscillations in 1/B. **a**, ${R}_{{xx}}$ of the W-layer as a function of ${V}_{b}$ and $B$ at charge neutrality and $T=0.3$ K (copy of Fig. 3a of the main text). The EI phase with diverging ${R}_{{xx}}$ expands with B-field. **b,c**, Line cuts of **a** at fixed ${V}_{b}=0.65$ V (red dashed line) and 0.67 V (yellow dashed line). The periodicity at high B-fields is half of that at low B-fields. The two-fold spin-valley degeneracy is removed by the Zeeman effect at high B-fields, thus doubling the periodicity. The hole density *p* was determined from the periodicity in 1/B, which is set by the hole Fermi surface.

The results above demonstrate transitions tuned by the magnetic field and pair density (controlled by *V*_b_) between the EI and QH states, which give rise to the observed quantum oscillations in the EI region of the phase diagram. *E*_b_ decreases continuously with pair density due to screening^27,29,46^, whereas *E*_c_ increases with *B*. When *E*_b_ becomes comparable to or smaller than *E*_c_, the EI transitions to layer-decoupled QH states when both layers are in the fully filled LLs^21,22^. The EI phase returns when the LLs are partially filled until the pair density exceeds the Mott density. Beyond the Mott density, excitons dissociate into an electron–hole plasma and only layer-decoupled LLs are present. In this picture, the frequency of the quantum oscillations in 1/*B* is set by the size of the electron–hole Fermi surface (Extended Data Fig. 8).

We perform mean-field calculations for the ground-state energy density of the electron–hole double layer^21^ to compare with experiment (Methods). The theoretical phase diagram (Fig. 3c) qualitatively captures the experimental phase diagram (Fig. 3a). A fan of layer-decoupled QH states at integer *ν*_e_ (=*ν*_h_) merges at high magnetic fields, and their onset field decreases with increasing *ν*_e_ or *ν*_h_. However, the scale of the magnetic field in the mean-field result is substantially higher because the density-dependent exciton binding energy due to screening and the associated exciton Mott transition have not been taken into account. Future studies including these effects are required to quantitatively describe the experimental results.

## Quantum oscillations in Coulomb drag

Finally, we perform the drag counterflow measurements to further examine the EI-to-QH transitions. We drive an a.c. bias current (*I*_drive_) in the Mo layer and measure the drag current (*I*_drag_) in the W layer^29^ (Fig. 1f), keeping interlayer tunnelling negligible (Extended Data Fig. 3). The drag ratio $\frac{{I}_{\rm{drag}}}{{I}_{\rm{drive}}}$ at low temperature, as shown for device 3 in Fig. 3b, provides a measure for the interlayer Coulomb coupling^29^. In particular, near-perfect Coulomb drag ($\frac{{I}_{\rm{drag}}}{{I}_{\rm{drive}}}\approx 1$) is observed for the EI phase; near-zero drag is observed for the layer-decoupled QH states (*ν*_e_ = *ν*_h_ = 2, 3 and 4) and the electron–hole plasma. The results are fully consistent with the phase diagram inferred from *R*_*xx*_ in Fig. 3a.

**Fig. 5:**
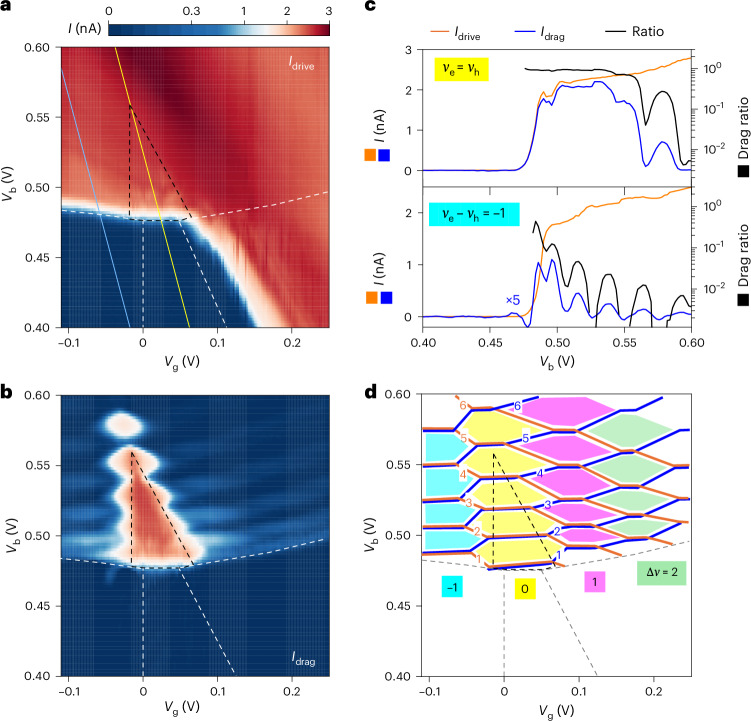
Finite-angular-momentum excitonic correlations. **a**,**b**, Drive current (*I*_drive_) in the Mo layer (**a**) and drag current (*I*_drag_) in the W layer (**b**) as a function of *V*_g_ and *V*_b_ at *B* = 12 T and *T* = 1.5 K. Near-perfect Coulomb drag is observed in the EI region shown schematically by the black dashed lines (weaker states above the triangle are not included at this temperature). Substantial Coulomb drag is also observed outside the EI region with partially filled LLs, indicating the presence of excitonic correlations with finite angular momentum. **c**, Line cuts in **a** and **b** (left axis) and drag ratio $\frac{{I}_{\rm{drag}}}{{I}_{\rm{drive}}}$ (right axis) along the solid yellow line with *ν*_e_ = *ν*_h_ (top) and cyan line with *ν*_e_ − *ν*_h_ = –1 (bottom) in **a**. An enhanced drag ratio indicates stronger interlayer excitonic interactions. The layer-decoupled QH states exhibit a substantial drop in the drag ratio. **d**, Phase diagram for the QH states in each layer (orange and blue lines for the Mo and W layers, respectively) constructed from **b** (see the main text). The regions with Δ*ν* = *ν*_e_ − *ν*_h_ = –1, 0, 1 and 2 are shaded in cyan, yellow, purple and green, respectively. The EI state dominates the Δ*ν* = 0 region. The dashed lines in **a**, **b** and **d** denote the boundaries of different doping regions from electrostatics and the EI from Coulomb drag. Source data

We further examine Coulomb drag in the presence of an electron–hole density imbalance. Figure 5a,b shows *I*_drive_ and *I*_drag_ in the Mo and W layers, respectively, as a function of *V*_g_ and *V*_b_ for device 2 at *B* = 12 T. The Mo layer is turned on electrically only in the i–n and p–n regions. A drag current in the W layer with nearly identical magnitude but opposite sign with respect to *I*_drive_ is observed in the triangular EI region. Quantum oscillations are observed in *I*_drag_ near the EI phase boundary and beyond. Figure 5c shows line cuts for the currents together with their ratio along the *ν*_e_ = *ν*_h_ and *ν*_e_ − *ν*_h_ = –1 lines (yellow and blue, respectively; Fig. 5a). Near-perfect Coulomb drag is observed at *ν*_e_ = *ν*_h_ for *V*_b_ ≲ 0.55 V; the drag ratio drops sharply and exhibits quantum oscillations with increasing *V*_b_. A large but imperfect drag (with ratio up to about 0.5) is observed at *ν*_e_ − *ν*_h_ = –1 near the onset of electron–hole injection; the drag ratio also drops sharply and shows quantum oscillations with increasing *V*_b_.

We construct a phase diagram in *V*_g_ and *V*_b_ for layer-decoupled QH states (*ν*_e_, *ν*_h_) by tracing zero drag (Fig. 5d). The regions with *ν*_e_ − *ν*_h_ = –1, 0, 1 and 2 are shaded. The EI with near-perfect Coulomb drag dominates the *ν*_e_ = *ν*_h_ region, except that near the tip of the triangle with *ν*_e_ − *ν*_h_ = 4, 5 and 6. The presence of substantial Coulomb drag (with ratio up to about 0.5) at partially filled LLs in the *ν*_e_ − *ν*_h_ = –1, 1 and 2 regions suggests the presence of interlayer excitonic correlations with angular momentum quantum of –*ℏ*, *ℏ* and 2*ℏ*, respectively^21^. The result suggests a tendency to stabilize EIs with finite-angular-momentum excitons, as suggested by mean-field calculations^21^. However, the imperfect Coulomb drag shows the presence of ionized electrons and holes possibly from thermal and/or quantum fluctuations. Further studies under higher magnetic fields and/or with stronger excitonic binding are required to fully establish the finite-angular-momentum EIs.

## Conclusion

We observe resistivity and Coulomb drag quantum oscillations in a dipolar EI based on Coulomb-coupled MoSe_2_/WSe_2_ electron–hole double layers. The oscillations originate from phase transitions between the competing EI and layer-decoupled QH states^21,22^. Our results demonstrate a highly tunable platform for studying quantum oscillations in correlated insulators and pave the way for explorations in several directions. To name a couple, the finite-angular-momentum excitonic correlations present a possibility to realize the QH effect for excitons. Studying how the collective excitations in the EI would evolve in a phase transition from the EI to QH states, in which there are only single-particle excitations, will provide insights into the nature of the phase transition.

## Methods

### Device design and fabrication

The device and contact geometry have been described in refs. ^27,29,32^. The optical images of devices 1 and 2 are shown in Extended Data Fig. 1a,b, respectively. The top and bottom graphite gates are outlined by the dashed and solid red lines, respectively. The WSe_2_ monolayer (green) and MoSe_2_ bilayer (blue) are separated by a thin hBN barrier (1.5–2 nm) in the transport channel (shaded red) and by a thick hBN barrier (10 nm) in the exciton contact regions (shaded pink). A natural MoSe_2_ bilayer is chosen because it does not crack as easily as a monolayer during the fabrication process. Under a high perpendicular electric field as used in this study, electrons in the bilayer are polarized to one layer and the bilayer functions effectively as a monolayer. To achieve high doping densities at the metal–semiconductor contacts for ohmic contacts at low temperatures, Pt–WSe_2_ contacts are gated only by the top gate; similarly, Bi–MoSe_2_ contacts are gated only by the bottom gate. During the measurements, *Δ* was maintained constant (3 V for device 1 and 5.5 V for device 2). Here $\varDelta \equiv \frac{{V}_{{{\rm{bg}}}}-{V}_{{{\rm{tg}}}}}{2}$ is the antisymmetric part of the top- and bottom-gate voltages *V*_tg_ and *V*_bg_, respectively; it is proportional to the perpendicular electric field (the symmetric part of the gate voltages, ${V}_{{\rm{g}}}\equiv \frac{{V}_{{{\rm{tg}}}}+{V}_{{{\rm{bg}}}}}{2}$, controls the net charge density in the channel).

**Extended Data Fig. 9:**
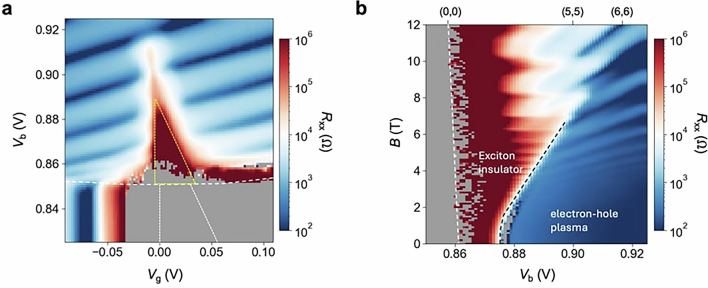
Quantum oscillations in device 3. **a**, ${R}_{{xx}}$ of the W-layer as a function of ${V}_{g}$ and ${V}_{b}$ at $B=12$ T and $T=1.5$ K. The triangular region enclosed by the yellow dashed lines is the EI region. The white dashed lines are the electrostatic phase boundaries at $B$ = 0 T. **b**, ${R}_{{xx}}$ as a function of ${V}_{b}$ and $B$ at charge neutrality (that is ${\nu }_{e}={\nu }_{h}$) and $T=1.5$ K. The EI boundary (black dashed line) expands with magnetic field. The fully filled LLs that protrude into the EI phase at high fields are identified as $\left({\nu }_{e},{\nu }_{h}\right)=\left(\mathrm{5,5}\right),\left(\mathrm{6,6}\right)$; the band edge (white dashed line) is labeled as $\left({\nu }_{e},{\nu }_{h}\right)=\left(\mathrm{0,0}\right)$. In the grey-shaded area, ${R}_{{xx}}$ diverges and cannot be reliably measured (**a,b**).

The devices were fabricated by the layer-by-layer dry transfer method^27,29,32,47^. In short, all the individual layers were first exfoliated by Scotch tape onto 285-nm SiO_2_/Si substrates and screened by an optical microscope for appropriate thickness and geometry before stacking. Layers were picked up sequentially using a polymer stamp made of a thin layer of polycarbonate on a polypropylene-carbonate-coated polydimethylsiloxane block. The completed stack was released onto pre-prepatterned Pt electrodes on SiO_2_/Si substrates to form contacts to the W layer and to the gate electrodes. The polymer residue was removed using chloroform and isopropanol. Next, separate contacts for the W layer were defined by etching the top graphite electrode using electron-beam lithography patterning (Nabity) and oxygen plasma reactive ion etching (Oxford PlasmaLab 80 Plus). Finally, contacts to the Mo layer were fabricated by another electron-beam lithography patterning followed by Bi evaporation in a thermal evaporator^48^. The main results have been reproduced in three devices. Extended Data Fig. 9 shows additional data from device 3.

### Electrical measurements

The transport properties of the electron–hole double layers were examined using two measurement configurations: the open-circuit *R*_*xx*_ and *R*_*xy*_ measurements (Fig. 1e) and the drag counterflow measurement (Fig. 1f), which reflect the charge and exciton transport properties, respectively^29,32^. Extended Data Fig. 2a,b shows the corresponding circuit diagrams. In the open-circuit geometry (device 1; Figs. 1–3), the Mo layer is kept in the open-circuit configuration, that is, no electron current (and, therefore, no exciton current) can flow; four-terminal *R*_*xx*_ and *R*_*xy*_ measurements are performed on the W layer using the standard lock-in technique. Here *R*_*xx*_ and *R*_*xy*_ measure the transport properties of the unbound holes in the W layer^32^. There are a total of eight contacts in the W layer defined by the etched top graphite gate (pins 1–8). To obtain *R*_*xx*_, a small a.c. bias voltage of 0.15 mV (root mean square (r.m.s.)) was applied between pins 2 and 6 at 11.33 Hz, and the bias current out of pin 6 and the longitudinal voltage drop between pins 1 and 5 were measured simultaneously. To obtain *R*_*xy*_, a small a.c. bias voltage of 0.15 mV (r.m.s.) was applied between pins 1 and 4 at 11.33 Hz, and the bias current out of pin 4 and the voltage drop between pins 2 and 6 were measured simultaneously. Other longitudinal and Hall measurement configurations gave similar results.

In the drag counterflow geometry (devices 2 and 3; Figs. 3 and 4), both layers are kept in the closed-circuit configuration so that excitons can flow in the transport channel. An a.c. bias current is driven in the Mo layer and the drag current is measured in the W layer (Fig. 1f). The drag ratio $\frac{{I}_{\rm{drag}}}{{I}_{\rm{drive}}}$ at low temperature reflects the exciton population fraction in the system^29^. In particular, $\frac{{I}_{\rm{drag}}}{{I}_{\rm{drive}}}=1$ and 0 correspond to pure exciton and pure charge transport, respectively. An a.c. bias voltage of 10 mV (r.m.s.) at 7.33 Hz was applied to the Mo layer through a 1:1 voltage transformer. The voltage transformer was connected to a 10-kΩ potentiometer to distribute the a.c. voltage on the two ends of the Mo layer; this step minimized the a.c. coupling between the Mo and W layers^42,43^. The d.c. interlayer bias voltage *V*_b_ was applied to the middle of the potentiometer; this kept both contacts in the Mo layer at the same d.c. potential and maintained a 10-mV a.c. voltage drop between them^29^. The drive current *I*_drive_ was measured by monitoring the voltage drop across a 150-kΩ resistor connected in series with the Mo layer; the drag current *I*_drag_ was measured by an ammeter connected in series with the W layer.

All the electrical measurements were carried out in a closed-cycle ^4^He cryostat (Oxford TeslatronPT) with temperature down to 1.5 K and magnetic field up to 12 T. All the currents and voltages were measured by lock-in amplifiers (Stanford Research Systems SR830 and SR860). In the voltage measurements, the signals were first sent to a preamplifier (Ithaco DL1201) with an input impedance of 100 MΩ before being measured by the lock-in amplifier. The measurement results are largely independent of the excitation amplitude (3–20 mV) and frequency (7–37 Hz).

### Capacitance measurements

Details of the capacitance measurements have been described in ref. ^27^. The penetration capacitance (*C*_P_) of the dual-gated devices 1 and 2 was measured under *B* = 12 T by applying an a.c. bias voltage of 5 mV (r.m.s.) at 737 Hz to the top gate and measuring the induced charge carrier density on the bottom gate by a GaAs high-electron-mobility transistor through a low-temperature capacitance bridge^49^. The resulting *C*_P_ as a function of *V*_g_ and *V*_b_ is shown in Extended Data Fig. 5. The exciton binding energy for each *V*_b_ (Fig. 4c) was obtained by integrating the normalized *C*_P_ with respect to *V*_g_ over a small window centred at charge neutrality, *E*_b_ ≈ ∫(*C*_P_/*C*_gg_)d*V*_g_, where *C*_gg_ is the gate-to-gate geometrical capacitance^27^.

### Mean-field calculations

To obtain the phase diagram shown in Fig. 3c, we performed mean-field calculations using the self-consistent Hartree–Fock approximation described in ref. ^21^, and assumed a uniform carrier distribution in our solutions. The single-particle part of the Hamiltonian consists of LLs from the two semiconductor layers; for simplicity, we assumed equal electron and hole cyclotron energies (a good approximation given the similar electron and hole masses of ~0.4*m*_0_, where *m*_0_ is the free electron mass). The LL energy in the conduction (valence) band increases (decreases) with the level index. The bias voltage *V*_b_ controls the effective energy difference between the conduction band minimum and the valence band maximum. We considered the screening effects from the gate electrodes on both intralayer and interlayer Coulomb interactions between charge carriers. In momentum (**q**) space, the intralayer and interlayer Coulomb interactions are ${V}_{{\rm{A}}}\left({\bf{q}}\right)=\frac{2\uppi {e}^{2}}{\epsilon q}\tanh ({qD})$ and *V*_E_(**q**) = *V*_A_(**q**)e^−*qd*^, respectively. The factor tanh(*qD*) comes from screening from the gate electrodes. Here *d* = 1.3*d*_0_ and *D* = 1.3*D*_0_ are the effective distances between the two semiconductor layers and between the two gates, respectively (*d*_0_ = 1.8 nm and *D*_0_ = 8 nm denoting the geometrical distances). The factor of 1.3 accounts for the anisotropic dielectric constant of hBN (*ε* ≈ 5.2). We also increased the dielectric constant by 50% to account for the correlated screening effect. Finally, we evaluated the Hartree contribution to the interaction as the potential energy of the capacitor and the exchange integrals following ref. ^21^.

## Online content

Any methods, additional references, Nature Portfolio reporting summaries, source data, extended data, supplementary information, acknowledgements, peer review information; details of author contributions and competing interests; and statements of data and code availability are available at 10.1038/s41563-025-02334-3.

## Source data

Source Data Fig. 1 *R*_*xx*_ at *B* = 0 T.

Source Data Fig. 2 *R*_*xx*_ and *R*_*xy*_ at *B* = 12 T.

Source Data Fig. 3 *R*_*xx*_, drag ratio along the p–n line versus the *B* field.

Source Data Fig. 4 *R*_*xx*_ and *R*_*xy*_ versus temperature.

Source Data Fig. 5 Drive current, drag current and drag ratio at *B* = 12 T.

## Data Availability

All data are available in the article. Source data are provided with this paper.
